# Differential Diagnosis of Orofacial and Jaw Pain: A Clinical Review for Physicians

**DOI:** 10.5041/RMMJ.10584

**Published:** 2026-10-26

**Authors:** Waseem A. Abboud, Tatiana Sella Tunis, Oren Peleg, Dror Shamir, Rania Elkhatib Fahoum, Shoshana Reiter, Michael V. Joachim

**Affiliations:** 1 Department of Oral and Maxillofacial Surgery, Goldschleger School of Dental Medicine, Gray Faculty of Medical and Health Sciences, Tel Aviv University, Tel Aviv, Israel; 2 Department of Neurology, Institute of Movement Disorders, Sheba Tel-Hashomer Medical Center, Ramat-Gan, Israel; 3 Unit of Oral and Maxillofacial Surgery, Bnai Zion Medical Center, Haifa, Israel; 4 Department of Orthodontics, Goldschleger School of Dental Medicine, Gray Faculty of Medical and Health Sciences, Tel Aviv University, Tel Aviv, Israel; 5 Department of Otolaryngology Head and Neck Surgery and Maxillofacial Surgery, Tel Aviv Sourasky Medical Center, Tel Aviv, Israel; 6 Department of Plastic Surgery, Bnai Zion Medical Center, Haifa, Israel; 7 Department of Oral Medicine, Goldschleger School of Dental Medicine, Gray Faculty of Medical and Health Sciences, Tel Aviv University, Tel Aviv, Israel; 8 Unit of Oral and Maxillofacial Surgery, Shamir (Assaf ha-Rofeh) Medical Center, Tzrifin, Israel; 9 Department of Plastic and Reconstructive Surgery, Shamir (Assaf ha-Rofeh) Medical Center, Tzrifin, Israel

**Keywords:** Clinical examination, differential diagnosis, facial pain, orofacial pain, physician education, temporomandibular joint

## Abstract

Orofacial and jaw pain represents a diagnostic challenge frequently encountered across multiple medical specialties. Although odontogenic disease is a common cause of pain in the facial region, the present review focuses on non-odontogenic causes that should be considered when no clear dental source is identified or symptoms remain unexplained or persist despite appropriate dental treatment. Such pain may originate from the temporomandibular joints, masticatory muscles, cranial nerves, facial skeleton, adjacent head-and-neck structures, systemic diseases, or distant referred sources. This comprehensive review presents a systematic clinical approach to differential diagnosis, classifying non-odontogenic orofacial pain into ten distinct categories: temporomandibular joint disorders, masticatory muscle pain, skeletal disorders of the jaws, space-occupying lesions, infectious processes, neuropathic orofacial pain, idiopathic and nociplastic pain, orofacial pain resembling a presentation of primary headache, systemic diseases with orofacial manifestations, and referred orofacial pain from distant medical conditions. This classification aligns with the International Classification of Orofacial Pain (ICOP-2020) while emphasizing practical clinical applicability for physicians across various specialties. Each category is characterized by unique clinical features, examination findings, and diagnostic considerations. Proper recognition of these patterns enables accurate diagnosis, appropriate treatment, and timely specialist referral when indicated. The approach emphasizes clinical assessment techniques accessible to physicians across various healthcare settings, focusing on detailed history-taking and physical examination. Identification of red flag symptoms requiring urgent evaluation is highlighted throughout. This systematic framework aims to reduce diagnostic errors, improve patient outcomes, and enhance interdisciplinary collaboration in managing complex orofacial pain presentations.

## INTRODUCTION

Orofacial and jaw pain represents a prevalent complaint encountered by physicians across a broad spectrum of medical specialties. Epidemiological data suggest that while dental pathology remains the most common cause of orofacial symptoms, a significant proportion of cases originate from non-dental structures, including the temporomandibular joint (TMJ), masticatory muscles, cranial nerves, and facial skeleton.^1^,^2^ A recent meta-analysis of the global prevalence of oral and facial pain estimated the pooled prevalence of concurrent oral and facial pain at approximately 19%, with oral pain reported in 21% and facial pain alone in 12% of the general population, underscoring the substantial global burden of these presentations.^3^

The diagnostic challenge for the non-dental clinician lies in the complex anatomy of the head and neck, where densely packed structures often refer pain to the teeth or jaws. Consequently, patients frequently misinterpret somatic or neuropathic pain as “toothache,” leading to potential delays in diagnosis or to unnecessary dental interventions. Physicians in primary care, neurology, rheumatology, ear, nose, and throat (ENT), and emergency medicine often serve as the first line of defense. However, a lack of familiarity with the clinical presentation of non-dental etiologies may lead to inappropriate referrals or failure to identify serious underlying pathology. For instance, vision-threatening conditions such as giant cell arteritis (GCA) may present initially as benign jaw claudication, mimicking temporomandibular disorders.^4^

To navigate this complexity, the clinician must identify the source of pain using primary clinical tools: accurate history-taking and a focused physical examination.^5^ The characteristics of the patient’s complaint—specifically the pain quality, timing, and response to functional activity (such as chewing and yawning)—are often sufficient to distinguish between musculoskeletal, neuropathic, and systemic causes. A symptom-driven approach to the initial assessment of non-odontogenic orofacial and jaw pain is summarized in Table 1.

**Table 1:** Symptom-driven Initial Assessment of Non-odontogenic Orofacial and Jaw Pain.

Presenting Pattern	Principal Possibilities	Suggested Disposition
Pain triggered by exertion or stress	Cardiac ischemia	Emergency cardiovascular evaluation
Age ≥50 with new headache or jaw claudication	Giant cell arteritis	Same-day ED or rheumatology
Acute painful trismus over hours to days	Deep-space infection; septic TMJ; trauma	Urgent hospital-based OMFS/ENT
Progressive restriction, asymmetry, deviation, or occlusal change	Ankylosis; coronoid hyperplasia; condylar growth; mass	Prompt OMFS/ENT referral
Preauricular pain, clicking, locking, or pain with jaw function	TMJ internal derangement; osteoarthritis; inflammatory arthritis	Conservative care; refer if persistent or locking
Diffuse jaw or facial pain with stiffness or fatigue	Masticatory myalgia; myofascial pain; sleep-related contributors	Conservative care; refer if persistent
Brief electric-shock pain triggered by touch, eating, or speaking	Trigeminal neuralgia; glossopharyngeal neuralgia	Neurology referral
Numbness, tingling, or sensory loss in V2/V3	Traumatic neuropathy; perineural spread; skull-base or CNS lesion	OMFS or neurology referral
Burning oral pain with normal-appearing mucosa	Burning mouth syndrome; secondary oral burning	Treat cause; oral medicine if persistent
Bilateral jaw pain with widespread pain, fatigue, or poor sleep	Fibromyalgia or nociplastic pain	Primary care or pain management
Ear fullness, tinnitus, or otalgia modified by jaw function	TMD/myalgia; primary otologic disease	ENT if otologic findings; otherwise TMD evaluation
Meal-related preauricular or submandibular pain or swelling	Sialolithiasis; duct stenosis; sialadenitis	ENT/OMFS referral
Cheek or maxillary pressure with nasal symptoms	Maxillary sinusitis, including odontogenic sinusitis	ENT or dental referral by suspected source
Recurrent pulsating facial or jaw pain with nausea or photo/phonophobia	Orofacial migraine	Headache management; neurology if atypical
Persistent poorly localized facial or dentoalveolar pain without findings	PIFP; PIDP; nociplastic pain	Orofacial-pain or oral medicine referral

Urgent red flags and escalation pathways are summarized in Table 2; selection of imaging modalities is presented in Table 3.

CNS, central nervous system; ED, emergency department; ENT, otolaryngology; OMFS, oral and maxillofacial surgery; PIDP, persistent idiopathic dentoalveolar pain; PIFP, persistent idiopathic facial pain; TMD, temporomandibular disorder; TMJ, temporomandibular joint.

This review presents a systematic clinical approach for differential diagnosis, aligned with the International Classification of Orofacial Pain (ICOP-2020),^6^ which provides the foundational taxonomy for orofacial pain conditions. To facilitate practical clinical decision-making for physicians across various specialties, we organize non-odontogenic orofacial pain into ten clinically relevant categories:

1. Temporomandibular joint disorders
2. Masticatory muscle pain
3. Skeletal disorders of the jaws and adjacent structures
4. Space-occupying and infiltrative lesions
5. Infectious processes in and adjacent to the masticatory system
6. Neuropathic orofacial pain
7. Idiopathic and nociplastic orofacial pain
8. Primary headache disorders presenting as orofacial pain
9. Systemic diseases with orofacial manifestations
10. Referred orofacial pain from distant medical conditions

This classification system aims to guide the physician toward targeted treatment, prevent diagnostic errors, and ensure timely referral to the appropriate specialist when indicated. It intentionally differs from the ICOP-2020 framework in both structure and clinical orientation; however, all conditions discussed can be accurately mapped to ICOP-2020 categories. The ICOP-2020 is a formal nosological system developed to provide precise diagnostic definitions and standardized terminology for orofacial pain specialists, primarily serving research, epidemiology, an expert-level clinical classification.^6^ In contrast, the present review is directed toward family physicians and non-dental medical specialists, for whom the primary clinical objective is early recognition, risk stratification, and appropriate referral rather than taxonomic precision. Key clinical red flags and recommended escalation pathways are summarized in Table 2. Guidance on the selection of imaging modalities according to the suspected clinical condition is provided in Table 3.

**Table 2:** Clinical Red Flags and Escalation Pathways in Orofacial and Jaw Pain.

Clinical Red Flag	Principal Concern(s)	Immediate Assessment	Action Pathway
Exertional jaw/facial pain, especially with chest discomfort, dyspnea, diaphoresis, nausea, or rest pain	Cardiac ischemia / acute coronary syndrome	Vital signs; ECG and troponin	EMERGENCY: immediate ED or cardiology
Age ≥50 with new headache or jaw claudication; visual or constitutional symptoms	Giant cell arteritis	Visual and temporal-artery exam; ESR, CRP, CBC	SAME-DAY: rheumatology or ED; do not delay steroids if strongly suspected
Sudden severe facial pain or headache with focal neurological deficit, altered consciousness, or progressive cranial-nerve dysfunction	Stroke or other acute intracranial process	Neurological exam; urgent CT/MRI	EMERGENCY: immediate ED or stroke evaluation
Unilateral head/face/neck pain with Horner syndrome, pulsatile tinnitus, or recent neck trauma/manipulation	Carotid or vertebral artery dissection	Neurological exam; urgent CTA or MRA	EMERGENCY: immediate ED or stroke service
Swallowing-triggered throat/ear pain with syncope, bradycardia, or hypotension	Glossopharyngeal neuralgia with cardioinhibitory reflex	Vitals, ECG/monitoring; neurological exam	EMERGENCY if syncope or hemodynamic compromise
Rapid painful trismus with fever, facial/neck swelling, dysphagia, muffled voice, drooling, or dyspnea	Deep facial/cervical infection; airway risk	Airway first; oral/neck exam; contrast CT	EMERGENCY: hospital OMFS/ENT; IV antibiotics ± drainage
Acute severe preauricular pain/swelling, fever, and marked restriction of jaw movement	Septic arthritis of the TMJ	CBC/CRP, cultures; CT/MRI; joint aspiration	SAME-DAY: ED/OMFS; antibiotics and drainage/lavage
Rapid salivary-gland swelling with fever, pus, severe tenderness, dehydration, or trismus	Suppurative sialadenitis or salivary abscess	Gland/duct exam; US ± contrast CT	SAME-DAY: ENT/OMFS; ED if toxic, spreading, or airway symptoms
Facial/maxillary pain with orbital swelling, visual loss, diplopia, proptosis, meningism, or neurological signs	Orbital or intracranial complication of sinusitis	Urgent ocular/neurological exam; CT/MRI	EMERGENCY: hospital with ENT/ophthalmology ± neurology
Otalgia/jaw pain with mastoid swelling, facial palsy, sudden hearing loss, severe vertigo, or diabetic/immunocompromised status	Complicated otitis, mastoiditis, or skull-base osteomyelitis	Otoscopy, cranial nerves; temporal-bone CT/MRI	SAME-DAY/EMERGENCY: urgent ENT or ED
Progressive trismus, new asymmetry/deviation, occlusal change, or unexplained preauricular/deep facial swelling	Ankylosis, skeletal lesion, or spaceoccupying mass	Jaw/occlusion/cranial-nerve exam; CT/MRI	EXPEDITED: OMFS/ENT; urgent if rapid, painful, or neurological
Persistent unilateral pain with ulcer/mass >2–3 weeks, induration, unexplained tooth mobility, dysphagia, neck mass, or weight loss	Head and neck malignancy	Full oral and head/neck exam; contrast imaging and biopsy	URGENT: OMFS/ENT oncology; avoid repeated empirical dental treatment
New or progressive facial/oral numbness, numb chin syndrome, facial weakness, or multiple cranial neuropathies	Perineural spread, metastasis, skull-base, or CNS lesion	Map deficits; cranial-nerve exam; contrast MRI ± CT	URGENT: OMFS/ENT and/or neurology; emergency if rapidly progressive
Trigeminal neuralgia with bilateral symptoms, sensory loss, young onset, continuous pain, or other neurological signs	Secondary neuralgia: MS, tumor, or structural lesion	Cranial-nerve exam; brain/trigeminal MRI	EXPEDITED: neurology; urgent if deficits progress
Persistent unilateral facial/jaw pain with smoking history, weight loss, cough, shoulder/arm pain, hand weakness, or Horner syndrome	Thoracic malignancy, including Pancoast tumor	Chest and neurological exam; chest CT	URGENT: respiratory or oncology assessment
Presumed TMD, primary headache, or idiopathic pain develops numbness, swelling, asymmetry, occlusal change, trismus, weight loss, or neurological decline	Missed structural, infectious, neoplastic, or neurological disease	Reopen differential; repeat exam; targeted tests/imaging	PROMPT–URGENT: stop irreversible treatment; redirect referral

Note: These findings warrant expedited, same-day, or emergency reassessment; local protocols take precedence.

CBC, complete blood count; CNS, central nervous system; CRP, C-reactive protein; CT, computed tomography; CTA, CT angiography; ECG, electrocardiography; ED, emergency department; ENT, otolaryngology; ESR, erythrocyte sedimentation rate; IV, intravenous; MRA, magnetic resonance angiography; MRI, magnetic resonance imaging; MS, multiple sclerosis; OMFS, oral and maxillofacial surgery; TMD, temporomandibular disorder; TMJ, temporomandibular joint; US, ultrasonography.

**Table 3:** Selection of Imaging Modalities in Non-odontogenic Orofacial and Jaw Pain.

Clinical Presentation or Suspected Condition	Initial Imaging?	Relevant Modalities Key	Points
*General screening across diagnostic categories*	*Consider after assessment*	*Panoramic radiography*	*Broad survey of teeth, jaws, and gross TMJ; targeted imaging may still be needed*
Typical masticatory myalgia or uncomplicated pain-related TMD	Usually no	None initially	Image for trauma, atypical or progressive signs, or non-response
TMJ internal derangement: painful clicking, locking, or persistent restriction	Usually no initially	TMJ MRI (open/closed mouth)	For persistent locking, uncertainty, or planned intervention
TMJ osteoarthritis or ankylosis	Yes if suspected	CBCT/CT ± MRI	CT for bone; MRI for inflammation or soft-tissue ankylosis
Progressive asymmetry, deviation, occlusal change, or skeletal lesion	Yes	Cephalometric radiographs; CBCT/CT; SPECT selectively	Define deformity or mass; SPECT may assess active bone growth
Eagle syndrome	Usually yes	Thin-section CT ± 3D	Imaging must correlate with the pain pattern
Space-occupying or infiltrative lesion	Yes; expedite if progressive	Contrast MRI ± CT/CBCT	MRI for soft tissue and nerves; CT for bone
Septic arthritis of the TMJ	Urgent	Contrast CT/MRI; joint aspiration	Do not delay antibiotics or drainage
Uncomplicated acute maxillary sinusitis	Usually no	Sinus CT selectively	For persistent, recurrent, complicated, or odontogenic disease
Deep facial or cervical space infection	Urgent	Contrast CT; MRI selectively	Airway and treatment take priority
Meal-related gland pain/swelling or salivary obstruction	Often	US; non-contrast or contrast CT	US for gland; CT for stones, abscess, or tumor
New-onset trigeminal neuralgia	Generally yes	High-resolution brain/trigeminal MRI	Assess compression and secondary causes
Progressive sensory loss, numb chin syndrome, or perineural spread	Urgent	Contrast MRI ± CT	Image the full nerve course; CT adds bone detail
Post-traumatic or iatrogenic trigeminal neuropathy	Selective	CBCT/CT ± MR neurography	Seek a reversible compressive cause
Stable orofacial migraine or primary headache	Usually no	CT/MRI only with red flags	Image red flags, not pain intensity
Persistent idiopathic facial/dentoalveolar pain, BMS, or nociplastic pain	No routine imaging	Targeted imaging only	Avoid repeat imaging without new objective signs
Inflammatory TMJ arthritis	Often	MRI ± CBCT/CT	MRI for active disease; CT for chronic damage
Giant cell arteritis	Supportive	Temporal/axillary artery US	Do not delay corticosteroids when suspicion is high
Carotid or vertebral artery dissection	Immediate	CTA or MRA ± brain MRI	Urgent stroke pathway
Thoracic source or Pancoast tumor	If suspected	Contrast chest CT ± MRI	With arm/shoulder symptoms, Horner syndrome, or weight loss

Note: Panoramic radiography is a broad screening survey, not a universal mandatory test. Each exposure should be clinically justified and tailored to the patient.

BMS, burning mouth syndrome; CBCT, cone-beam CT; CT, computed tomography; CTA, CT angiography; MRA, magnetic resonance angiography; MRI, magnetic resonance imaging; SPECT, single-photon emission CT; TMD, temporomandibular disorder; TMJ, temporomandibular joint; US, ultrasonography.

Accordingly, pain conditions are organized here by anatomical source, clinical presentation, and diagnostic workflow rather than by a strict etiological or pathophysiological hierarchy. This pragmatic structure reflects real-world clinical reasoning, where rapid pattern recognition and differentiation between articular, muscular, skeletal, neuropathic, systemic, and referred pain are essential. This framework is therefore intended to bridge formal ICOP-2020 classification and everyday clinical practice, serving as a complementary, clinically intuitive tool rather than an alternative nosological system. A rapid clinical triage algorithm for the initial evaluation of patients presenting with orofacial and jaw pain is shown in Figure 1.

**Figure 1:**
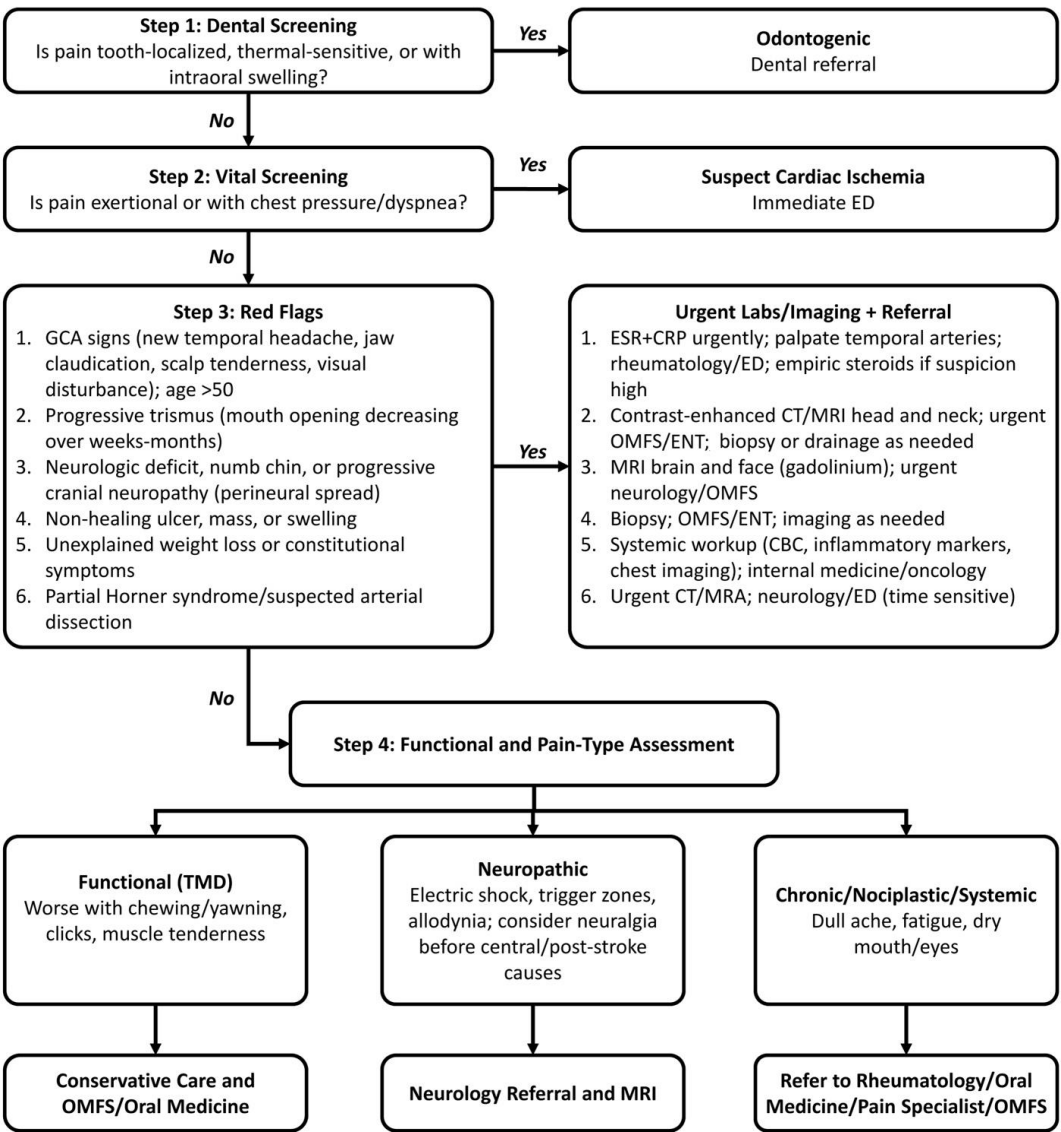
Rapid Clinical Triage Algorithm for Initial Evaluation of Orofacial and Jaw Pain in Primary-care and Emergency Settings. CBC, complete blood count; CRP, C-reactive protein; CT, computed tomography; ED, emergency department; ENT, ear, nose, and throat; ESR, erythrocyte sedimentation rate; GCA, giant cell arteritis; MRA, magnetic resonance angiography; MRI, magnetic resonance imaging; OMFS, oral and maxillofacial surgery; TMD, temporomandibular disorder.

## METHODS

This article is a narrative clinical review rather than a systematic review, intended to provide practicing physicians with a pragmatic diagnostic framework rather than an exhaustive quantitative synthesis. The literature was identified through searches of PubMed/MEDLINE, Scopus, and Google Scholar from database inception through 2025. Search terms included combinations of “orofacial pain,” “facial pain,” “jaw pain,” “temporomandibular disorders,” “trigeminal neuralgia,” “neuropathic facial pain,” “referred orofacial pain,” and “differential diagnosis,” together with terms specific to the individual conditions discussed in each section. Priority was given to peer-reviewed English-language publications, with an emphasis on systematic reviews, meta-analyses, consensus statements, clinical practice guidelines, and authoritative reference texts. Additional sources were identified by screening the reference lists of relevant papers. Because the aim was clinical utility for a non-specialist medical readership, studies were selected on the basis of clinical relevance, methodological soundness, and applicability to real-world diagnostic reasoning rather than through a formal protocol-driven or quality-scoring process. The selected evidence was then synthesized and organized into ten clinically oriented categories. This review does not claim to be exhaustive, and some relevant publications may not have been captured.

## 1. TEMPOROMANDIBULAR JOINT DISORDERS

Temporomandibular joint disorders are among the most common causes of non-dental orofacial pain. Epidemiological studies estimate the prevalence of temporomandibular disorder (TMD)-related pain in the adult population at approximately 10%–12%, with a female-to-male predilection of 2:1.^1^,^7^ These conditions encompass internal derangement of the TMJ and degenerative joint disease/TMJ osteoarthritis. 8 In addition, inflammatory TMJ arthritides include rheumatoid arthritis, juvenile idiopathic arthritis, spondyloarthropathies such as psoriatic arthritis, and crystal-induced arthropathies such as gout and pseudogout, which are discussed under section 9 of this review (Systemic Diseases with Orofacial Manifestations).

### Clinical Presentation

The typical complaints and findings presented by the patient with an articular disorder include: sounds during TMJ movement, which can be described as “clicks” or “cracks” in cases of internal derangement, or “grinding” and “bone friction” in cases of arthritis; pain localized to the ear, or preauricular, which the patient often points to with one finger and not with the whole hand, usually worsening during chewing or yawning;^7^ limitation in mouth opening, which can be transient or permanent; jaw deviation during opening or protrusive movement toward the affected/painful side with or without straightening and return to center, with possible limitation in lateral movement to the opposite side;^9^ and tenderness in the TMJ area or preauricular area during various functional tests.

Familiarity with the Wilkes classification system of internal derangement (stages I through V) aids the physician in clinical assessment, without necessarily classifying the patients according to it:^10^ I, benign clicks in the TMJ; II, clicks and temporary locks and/or momentary pain; III, acute development of permanent limitation in mouth opening; IV, prolonged/ chronic limitation in mouth opening; and V, development of degenerative changes on top of prolonged internal derangement.

Regarding degenerative diseases such as osteoarthritis, classifications based on computed tomography (CT) or magnetic resonance imaging (MRI) divide findings into normal, mild, moderate, or advanced changes. Hence, final diagnosis may require advanced imaging such as CT or MRI.

### Initial Management

Management should begin with conservative measures, including a soft diet, limitation of wide mouth opening, and short-term use of non-steroidal anti-inflammatory drugs (NSAIDs) if no contraindications exist. Patients should be educated on avoiding parafunctional habits such as gum chewing and nail biting. If symptoms persist beyond 2–3 weeks or if locking occurs, referral to an oral and maxillofacial surgeon, a specialist in oral medicine, or a dentist with TMD expertise is indicated.

## 2. MASTICATORY MUSCLE PAIN

Disorders of muscular origin are the most common temporomandibular cause of facial and jaw pain.^11^,^12^ Muscular disorders can be classified as local myalgia, myofascial pain (localized and referring), muscle spasm, myositis, and myofibrotic contracture.^13^

### Clinical Presentation

The typical complaints include: feeling of stiffness or fatigue in the jaws and face; dull and spreading pains, less localized compared to articular pain, where the patient typically indicates the area with the whole hand rather than one finger;^14^ tenderness on palpation of masticatory muscles; sometimes accompanying phenomena such as ear fullness and tinnitus, though ENT pathology should first be excluded; and in examining muscle hardening during teeth clenching, either hyper-contraction with abnormal stiffness or paradoxical weakness may be noted.^15^

The diagnosis of masticatory muscle problems is usually based on history and physical examination alone, with imaging performed to rule out other conditions. Masticatory muscle pain may also occur as part of systemic nociplastic disorders such as fibromyalgia (see section 7, Idiopathic and Nociplastic Orofacial Pain). It is also frequently associated with sleep-related conditions, particularly sleep bruxism, defined as repetitive masticatory muscle activity during sleep. Sleep-disordered breathing may also indirectly precipitate or perpetuate masticatory myalgia and morning jaw pain^12^ (see Sleep Disorders and Orofacial Pain, below).

### Initial Management

First-line treatment focuses on self-care and behavioral modification, including applying moist heat to affected muscles, soft diet, and stress reduction techniques. Pharmacological management may include muscle relaxants (e.g. benzodiazepines, baclofen, cyclobenzaprine) or short-term NSAIDs. Evaluation of nocturnal bruxism is essential, and an occlusal splint (night guard) may be required. Referral to a specialist for physical therapy or trigger point injections may also be recommended.

## 3. SKELETAL DISORDERS OF THE JAWS AND ADJACENT STRUCTURES

Disorders of skeletal origin in the facial bones are an uncommon cause of orofacial pain, but recognition is important due to accompanying structural and functional implications.^16^ This heterogeneous group includes five main conditions, as detailed below.

### Condylar Hyperplasia

Condylar hyperplasia is characterized by excessive or prolonged growth of the mandibular condyle, sometimes involving the entire affected hemimandible. It causes progressive facial asymmetry, deviation of the chin and mandible, and changes in dental occlusion. The etiology is usually unknown, although hormonal, vascular, traumatic, and genetic factors have been proposed.^16^–^18^

The resulting skeletal and occlusal asymmetry may impair normal masticatory function by producing an uneven bite and altered mandibular movement. Aside from jaw deviation, patients may report difficulty chewing, jaw fatigue, joint sounds, irregular mandibular movements, and pain in the TMJ or masticatory muscles. However, some patients have marked facial and occlusal deformity with little or no pain.

When condylar hyperplasia is suspected, the patient should be referred to an oral and maxillofacial surgeon for assessment of disease activity and skeletal deformity; treatment is generally surgical and includes a form of condylectomy to stop the hyperplastic growth causing jaw and occlusal deviation.

### Temporomandibular Joint Ankylosis

Temporomandibular joint ankylosis is a pathological fusion of the articular surfaces of the joint, which may be fibrous, bony, or a combination of both, resulting in abnormal attachment of the mandibular condyle to the skull base. The most common causes are trauma, particularly condylar fractures, and infection involving the TMJ, historically arising from otitis media or mastoiditis.^19^–^21^

Clinically, TMJ ankylosis manifests as a persistent mechanical restriction of mouth opening, with reduced mandibular movements and a firm end feel. Unilateral ankylosis may cause deviation of the mandible toward the affected side, facial asymmetry, reduced mandibular growth on that side, and malocclusion. Bilateral ankylosis may result in mandibular hypoplasia, retrognathia, and the characteristic “bird-face” deformity.

Suspected TMJ ankylosis requires prompt referral to an oral and maxillofacial surgeon, with treatment generally involving surgical release of the ankylotic mass, followed by early and prolonged physiotherapy.

### Coronoid Process Hyperplasia

This disorder manifests with mechanical limitation in mouth opening due to elongation of the mandibular coronoid and its collision with the zygoma (cheekbone). It usually presents without pain and without external structural distortion or facial asymmetry. The cause is unknown.^22^,^23^

Patients with suspected coronoid hyperplasia should be referred to an oral and maxillofacial surgeon, as definitive treatment usually consists of unilateral or bilateral coronoidectomy, followed by intensive postoperative physiotherapy to restore and maintain mouth opening.

### Skeletal Deformity Secondary to Juvenile Idiopathic Arthritis

This systemic rheumatic disease can affect the TMJ, manifesting as posterior mandibular position with or without deviation, sometimes accompanied by mechanical limitation in mouth opening.^24^,^25^ Because of the prominent skeletal changes it produces, it is mentioned under this category rather under section 9 (Systemic Diseases with Orofacial Pain Manifestations).

In addition to appropriate rheumatologic therapy, patients with juvenile idiopathic arthritis-related TMJ involvement should be referred early for coordinated orthodontic and oral and maxillofacial evaluation; treatment may include growth modification, orthodontic correction, and corrective orthognathic surgery. Severe joint destruction and deformity may require total TMJ reconstruction.

### Eagle Syndrome

Eagle syndrome results from an elongated styloid process or a calcified stylohyoid ligament and may produce referred facial, cervical, and pharyngeal pain through irritation of adjacent cranial nerves and surrounding soft tissues. Patients typically report unilateral throat or facial pain, a foreign-body sensation, dysphagia, and otalgia, often aggravated by head turning or swallowing. Because these symptoms overlap with glossopharyngeal neuralgia, TMD, and other orofacial pain syndromes, the diagnosis is frequently delayed. Reproduction of pain on palpation of the tonsillar fossa is suggestive, and the diagnosis is confirmed by imaging demonstrating an elongated styloid process, best seen on CT with three-dimensional reconstruction.^26^,^27^

Each of these five conditions may present with varying degrees of pain and masticatory-system dysfunction, ranging from mild to severe. Each is associated with structural changes in the jaws or TMJ and typically produces recognizable radiographic findings, often detectable on initial panoramic imaging. Once one of these conditions is suspected, the patient should be referred to an oral and maxillofacial surgeon for comprehensive assessment, as definitive diagnosis usually requires advanced imaging with CT, MRI, or bone scans, and treatment is frequently surgical. Early referral is important because intervention before severe functional limitation, asymmetry, or skeletal deformity develops is generally less complex and is associated with superior functional and facial outcomes.

## 4. SPACE-OCCUPYING AND INFILTRATIVE LESIONS

Space-occupying and infiltrative lesions affecting the jaws, TMJ, and adjacent deep facial spaces include benign neoplasms and tumor-like proliferations, primary and metastatic malignancies, and hematologic infiltrative diseases. Because many arise in regions not accessible to direct clinical examination, they may initially present with persistent pain, progressive trismus, facial asymmetry, occlusal change, or other complaints resembling TMD. Although uncommon, these lesions are clinically important because their non-specific presentation may delay diagnosis, particularly in patients with neoplastic disease.^28^,^29^

### Benign Space-occupying Lesions

Benign space-occupying lesions of the mandibular condyle and TMJ are uncommon but clinically important because their slow growth and non-specific presentation may lead to prolonged treatment for presumed TMD. Osteoma and osteochondroma of the mandibular condyle typically produce gradual unilateral condylar enlargement and may present with progressive facial asymmetry, chin deviation, altered dental occlusion, restricted mandibular movement, or preauricular pain. Osteoma generally appears as a well-circumscribed osseous mass, whereas osteochondroma is an exophytic, cartilage-capped bony lesion that may substantially alter the morphology of the condyle and ramus.^30^–^32^ Computed tomography (CT) or cone-beam CT is particularly useful for defining the osseous morphology and relationship of the lesion to the skull base, although definitive diagnosis may require histopathological examination.

Synovial chondromatosis is a benign proliferative disorder of the synovium characterized by the formation of cartilaginous nodules that may detach and form loose bodies within the joint. Involvement of the TMJ commonly presents with unilateral preauricular pain or swelling, crepitus, progressive limitation of mouth opening, and deviation during mandibular movement, thereby closely resembling internal derangement or degenerative joint disease.^31^,^32^ A CT scan may demonstrate calcified or ossified loose bodies and expansion of the joint space, whereas MRI is more sensitive for detecting non-mineralized nodules, synovial proliferation, joint effusion, and extension into adjacent structures.

Benign tumors and tumor-like lesions may also arise outside the TMJ within the infratemporal, pterygopalatine, parapharyngeal, and other deep facial spaces.^28^ These include peripheral nerve-sheath tumors, lipomatous and other mesenchymal tumors, and benign lesions arising from adjacent salivary, vascular, or skeletal tissues. Their deep location makes them difficult to detect clinically because they may not produce a visible or palpable mass until they become relatively large. Initial manifestations may therefore be non-specific and include persistent unilateral pain, progressive trismus, facial fullness or asymmetry, altered occlusion, dysphagia, or sensory or motor disturbance caused by compression of adjacent structures. Unexplained progressive symptoms should prompt cross-sectional imaging, particularly contrast-enhanced CT or MRI, and referral for specialist evaluation.

### Malignant Neoplasms and Cancer-related Orofacial Pain

Head and neck malignancies—most commonly squamous cell carcinoma of the oral cavity, oropharynx, and nasopharynx, but also salivary gland, sinonasal, and skull-base tumors—are an important and frequently underrecognized cause of orofacial pain, and pain may be the first or only symptom. Because malignant pain can imitate virtually any orofacial pain disorder, presenting as a toothache, gingival pain, temporomandibular-type pain, or trigeminal neuralgia, cancer must be considered in the differential diagnosis of any unexplained, persistent, or treatment-resistant orofacial pain.^33^ Pain that is unilateral, progressive, unremitting, worse with function, and poorly responsive to conventional analgesics or dental treatment is particularly suspicious. Local warning signs include a non-healing ulcer or mucosal lesion persisting beyond two to three weeks, an indurated or exophytic mass, a tooth mobility or loss unexplained by tooth decay or periodontal disease, and sensory disturbance of the face and mouth.

### Perineural Invasion and Spread

Perineural invasion is a hallmark of several head and neck cancers—particularly squamous cell carcinoma and adenoid cystic carcinoma—and is reported in a substantial proportion of cases, with the trigeminal and facial nerves most often affected.^34^ Tumor tracking along nerves produces neuropathic orofacial pain (burning, tingling, or electric shock-like) accompanied by progressive sensory loss or motor deficits in the distribution of the involved nerve. The combination of facial pain with an evolving cranial neuropathy—especially progressive numbness, paresthesia, or weakness—should raise strong suspicion for perineural spread and prompts imaging (e.g. gadolinium-enhanced MRI of the skull base and cranial nerves).^33^

### Metastatic and Hematologic Disease

Distant malignancies may metastasize to the jaws, the TMJ, or the oral soft tissues; the breast, lung, prostate, kidney, and thyroid are the most frequent primary sites, and in a large proportion of cases the oral metastasis is the first evidence of an occult primary tumor.^35^ Metastatic deposits in the posterior mandible characteristically present with pain, swelling, and sensory disturbance, and TMJ pain may occasionally be the first symptom of metastasis. Hematologic disease, including multiple myeloma, lymphoma, and leukemia, can infiltrate the jaws and produce bone pain, swelling, tooth mobility, or root resorption.^29^ Numb chin syndrome (mental nerve neuropathy) is a particularly ominous neurologic sign in this setting and is frequently associated with metastatic or lymphoproliferative disease.

### Red Flag Symptoms

Clinicians must recognize the following red flags: jaw deviation or facial structure change developing after growth completion; gradual change in dental occlusion not explained by periodontal disease or normal wear; progressive limitation in mouth opening over weeks to months;^32^ difficulty swallowing or neck fullness; and sensory disturbance in facial or oral areas indicating possible nerve compression.

A particularly ominous sign is numb chin syndrome (mental nerve neuropathy); studies indicate that 30%–50% of cases are associated with metastatic malignancy (e.g. breast, lung, prostate) or lymphoproliferative diseases.^36^

### Diagnostic Approach

The physician serves as critical first line for diagnosis. Rapid referral to oral and maxillofacial surgery or ENT medicine may fundamentally affect disease prognosis and patient survival. Final diagnosis requires advanced imaging: CT, positron emission tomography–computed tomography (PET-CT), MRI, ultrasonography (US), or bone mapping.

## 5. INFECTIOUS PROCESSES IN AND ADJACENT TO THE MASTICATORY SYSTEM

Infectious conditions of the head and neck may present with facial or jaw pain and can closely mimic TMJ or masticatory muscle disorders, particularly in early stages. Failure to recognize an infectious etiology may result in delayed diagnosis and progression to serious local or systemic complications. In contrast to musculoskeletal pain, infectious pain is typically characterized by progressive intensity and limited response to functional rest. The most clinically relevant entities in this category include paranasal sinus infections, otologic infections, and infections involving the deep facial spaces.

### Maxillary Sinusitis

Maxillary sinusitis may be acute or chronic and is most commonly of viral or bacterial origin.^2^,^37^,^38^ In a subset of cases, it is odontogenic, arising from infection of maxillary posterior teeth, periapical pathology, or advanced periodontal disease. Patients typically describe a dull, pressure-like pain in the cheek, infra-orbital region, or maxillary posterior teeth, often accompanied by a sensation of fullness beneath the eye. A key distinguishing feature is exacerbation of pain with forward head flexion or positional changes, while pain generally does not increase with chewing, jaw movement, or mouth opening—features that help differentiate sinus-related pain from TMD.^37^,^38^ Associated symptoms such as unilateral nasal discharge, nasal congestion, hyposmia, or postnasal drip—when present—guide the clinician toward a diagnosis of sinusitis rather than a TMD. On examination, tenderness may be elicited over the affected maxillary sinus. Diagnosis is primarily clinical, with imaging reserved for persistent, atypical, or treatment-resistant cases. Computed tomography of the paranasal sinuses is the imaging modality of choice to demonstrate mucosal thickening, fluid levels, or obstruction of the ostiomeatal complex. When an odontogenic source is suspected, targeted dental evaluation is mandatory.

### Otologic Infections

Otologic infections, particularly acute otitis media and otitis externa, may present with pain perceived in the temporomandibular region or posterior mandible due to shared sensory innervation. Patients often report deep, unilateral ear pain radiating toward the jaw or preauricular area, sometimes exacerbated by pressure on the tragus or manipulation of the auricle rather than by mastication. The presence of associated otologic symptoms—such as ear fullness, hearing impairment, tinnitus, otorrhea, or fever—facilitates clinical differentiation from TMD. Otoscopic examination is essential for diagnosis. Jaw function is generally preserved, and limitation of mouth opening, if present, is mild and secondary to pain rather than mechanical restriction. When otologic infection is suspected, prompt referral to an otolaryngologist is indicated. Failure to identify an ear-related source may lead to unnecessary dental or temporomandibular interventions.^39^–^41^

### Septic Arthritis of the TMJ

Septic arthritis of the TMJ is a rare but potentially destructive infection that may result from hematogenous spread, contiguous extension from an adjacent odontogenic, otologic, or deep-space infection, or direct inoculation following trauma or an invasive procedure. Patients typically present with acute or subacute unilateral preauricular pain and tenderness, marked limitation of mouth opening, and pain during mandibular movement. Preauricular swelling and sudden alteration of the dental occlusion may also occur.^42^,^43^ Fever and systemic symptoms support the diagnosis but may be absent; therefore, the combination of rapidly progressive TMJ pain and trismus should raise suspicion even in an afebrile patient.

Initial evaluation should include examination for preauricular swelling, joint tenderness, mandibular deviation or new malocclusion, and possible dental, otologic, or deep facial sources of infection. Laboratory testing commonly includes a complete blood count and inflammatory markers, with blood cultures when systemic infection is suspected. Contrast-enhanced CT can demonstrate joint-space widening, effusion, osseous changes, and extension into adjacent spaces, whereas MRI is more sensitive for early joint effusion, synovitis, marrow involvement, and periarticular soft-tissue spread. Definitive diagnosis generally requires joint aspiration, with synovial fluid submitted for Gram staining, culture, and antimicrobial susceptibility testing.

Suspected TMJ septic arthritis requires urgent referral for hospital-based management. Treatment consists of prompt systemic antibiotic therapy together with drainage of the infected joint by arthrocentesis, arthroscopy, or open surgery, depending on the extent of infection and response to initial treatment. Any associated odontogenic, otologic, or deep-space source must also be treated. Once the acute infection is controlled, early mandibular mobilization and physiotherapy are important to restore function and reduce the risk of fibrous adhesions or ankylosis. Delayed diagnosis may result in cartilage and bone destruction, osteomyelitis, persistent joint dysfunction, ankylosis, sepsis, or—particularly in children—disturbance of mandibular growth.

### Sialadenitis and Obstructive Salivary Disease

Sialadenitis and obstructive salivary-gland disease may produce pain in the preauricular, submandibular, or posterior mandibular regions and may therefore be mistaken for temporomandibular or masticatory muscle pain. Obstruction is most commonly caused by a salivary stone or ductal stenosis and frequently affects the submandibular gland. Patients typically report recurrent pain and swelling that worsen during meals, when salivary flow increases against the obstruction.^44^ Secondary bacterial infection may cause persistent swelling, marked tenderness, erythema, fever, and purulent discharge from the involved duct. Acute suppurative parotitis generally presents with painful preauricular swelling, sometimes accompanied by trismus or pain during mastication.

Clinical examination should include inspection and bimanual palpation of the major salivary glands and their ducts, with attention to glandular enlargement, tenderness, reduced salivary flow, a palpable stone, or purulent drainage from the parotid or submandibular duct orifice. Unlike TMD, symptoms are more closely related to meals and glandular swelling than to jaw loading or mandibular movement. Ultrasonography is often the preferred initial imaging modality for suspected sialolithiasis or glandular inflammation, while non-contrast CT is more sensitive for small or deeply located calculi. Contrast-enhanced CT is indicated when abscess formation, deep extension, or another complication is suspected. Management depends on the cause and severity and may include hydration, gland massage, sialogogues, analgesia, and appropriate antibiotics when bacterial infection is present. Persistent obstruction, recurrent infection, suspected abscess, or progressive swelling warrants referral to an otolaryngologist or oral and maxillofacial surgeon.

### Deep Facial Space Infections

Infections involving the deep facial and cervical spaces—including the pterygomandibular, submasseteric, infratemporal, and parapharyngeal spaces—constitute a medical emergency.^45^,^46^ These infections most commonly arise as complications of odontogenic infections or soft-tissue trauma and are not accessible to direct palpation. Patients typically present with progressive unilateral pain that worsens with jaw function and is accompanied by marked limitation in mouth opening (trismus). The presence of additional features such as dysphagia, odynophagia, voice changes, neck stiffness, fever, and systemic inflammatory signs further raises suspicion for deep space involvement. The clinical course is often aggressive, with deterioration over days rather than weeks. Superficial findings, including facial swelling, may be minimal or absent despite extensive deep infection. Definitive diagnosis requires contrast-enhanced CT of the head and neck to delineate abscess formation, extent of infection, and involvement of adjacent vital structures. Management mandates immediate systemic antibiotic therapy and, in many cases, urgent surgical drainage. The critical role of the primary physician is early recognition of the atypical pain pattern and rapid referral to hospital-based care.

## 6. NEUROPATHIC OROFACIAL PAIN

Neuropathic pain arises from lesion or disease affecting the somatosensory nervous system.^6^ Unlike nociceptive pain, these disorders usually follow a neuroanatomically plausible distribution and may be accompanied by positive or negative somatosensory abnormalities.

### Clinical Characteristics

Neuropathic pain is defined by several key indicators: quality (sharp, shooting, electric-shock-like in neuralgias, or chronic continuous burning); sensory abnormalities (hypersensitivity to light touch/allodynia, or paradoxical combination of hyperesthesia and hypoesthesia); and pharmacological response (failure to respond to conventional analgesics such as NSAIDs or acetaminophen).

Diagnosis relies on accurate history-taking and detailed sensory examination. For suspected neuralgia or central involvement, brain and brainstem MRI is the gold standard to rule out space-occupying lesions or demyelinating disease (e.g. multiple sclerosis). For suspected peripheral nerve injury, nerve conduction study may confirm significant damage, though normal results do not rule out small-fiber neuropathy.

#### Paroxysmal Cranial Neuralgias

##### Trigeminal Neuralgia

Trigeminal neuralgia is characterized by episodic neuropathic facial pain. The majority of cases arise from neurovascular compression of the trigeminal root; some are idiopathic, and a minority are secondary to space-occupying lesions or multiple sclerosis.^47^ Clinical features include unilateral electric shock-like pain lasting seconds to minutes, trigger zones, pain-free intervals between attacks, and absence of neurological deficits in classical cases. Diagnosis is primarily clinical; however, MRI is required to determine etiology (neurovascular compression, idiopathic, or secondary).

Multiple sclerosis is a demyelinating disease typically appearing between ages 20 and 40. One prominent orofacial manifestation is secondary trigeminal neuralgia, clinically resembling classical neuralgia but potentially presenting bilaterally—a rare pattern that should always raise suspicion for a central disease.^48^,^49^ When a young patient presents with neuralgia, especially if bilateral, suspicion of demyelinating disease should be high, requiring neurological referral.

##### Glossopharyngeal Neuralgia

This is a relatively rare neuropathic condition caused by disruption of cranial nerve IX function, which innervates the pharynx, base of tongue, and part of inner ear. Glossopharyngeal neuralgia is characterized by brief severe pain in the ear, base of tongue, tonsillar region, or beneath the angle of the jaw, often triggered by swallowing, talking, coughing, or yawning.^50^ Rare attacks may be accompanied by bradycardia or syncope because of vagal reflex activation.

#### Painful Trigeminal Neuropathies

##### Post-traumatic/Iatrogenic Trigeminal Neuropathic Pain

Although dental procedures such as extraction, implant placement, and surgical interventions are usually uneventful, rare cases of nerve fiber injury may lead to persistent neuropathic pain.^51^,^52^ Clinical presentation includes sensory deficits (hypoesthesia, paresthesia, dysesthesia), burning or shooting pain in affected nerve distribution, allodynia, hyperalgesia, and phantom sensations.

##### Post-herpetic Neuralgia

Varicella zoster virus (VZV) reactivation causes shingles (herpes zoster). In most cases pain subsides after rash healing. However, in a minority of patients, it persists as neuropathic pain for months, known as post-herpetic neuralgia. By definition, pain lasting more than 3 months in the distribution of a trigeminal branch previously affected by herpes zoster is classified as trigeminal post-herpetic neuralgia.^53^

#### Central Neuropathic Pain

##### Central Post-stroke Facial Pain (Brainstem Infarction)

Focal ischemic damage in the brainstem, especially in the trigeminal nerve root entry zone or sensory nucleus, may lead to sudden facial pain or headache, sometimes in sharp neuralgia-like form, without prominent neurological deficit. Because the lesion lies within the central somatosensory pathways, the pain is central (neurogenic) in origin, resulting from disruption of pain transmission or sensory regulation in the central nervous system rather than from a peripheral nerve injury. Symptoms typically appear abruptly and are continuous, with additional neurological signs often emerging within hours.^54^,^55^ When stroke is suspected, immediate neurological examination, assessment using stroke scales, urgent neuroimaging with CT or MRI, neurology consultation, and time-sensitive intervention protocols are essential.

Other than the acute presentation, chronic neuropathic orofacial pain may develop as a sequel of a lesion in the central somatosensory pathways. This is often called central post-stroke facial pain.

##### Central Neuropathic Pain Attributed to Multiple Sclerosis

While multiple sclerosis can produce secondary trigeminal neuralgia (brief, triggered, electric-shock-like attacks attributable to a demyelinating lesion affecting trigeminal pathways), it can also produce central neuropathic pain. This manifests with variable continuous or intermittent craniocervical pain, sometimes accompanied by sensory changes. Overall, multiple-sclerosis-related orofacial symptoms include bilateral trigeminal neuralgia, constant facial pain, facial numbness, altered sensation, facial weakness, speech and swallowing difficulties, and dental issues secondary to facial numbness.^48^,^49^

### Initial Management of Neuropathic Pain

Recognition of neuropathic pain is essential, as it often responds poorly to conventional analgesics such as NSAIDs or acetaminophen, although treatment response alone should not be used to establish the diagnosis. Initial management should be guided by the clinical phenotype, underlying cause, comorbidities, and severity of symptoms.

For trigeminal neuralgia, first-line pharmacological treatment is carbamazepine or oxcarbazepine. Similar sodium-channel-blocking agents may also be considered for glossopharyngeal neuralgia under appropriate specialist supervision.^47^ New-onset trigeminal neuralgia generally warrants brain MRI to investigate secondary causes and assess for neurovascular compression. Patients with atypical features, bilateral symptoms, sensory deficits, young age at onset, abnormal imaging, diagnostic uncertainty, or inadequate response to treatment should be referred to a neurologist or other appropriately experienced specialist. Refractory cases may require neurosurgical evaluation.

Continuous painful trigeminal neuropathies, including painful post-traumatic trigeminal neuropathy and trigeminal post-herpetic neuralgia, are generally treated with medications used for neuropathic pain, such as tricyclic antidepressants, serotonin–norepinephrine reuptake inhibitors, gabapentin, or pregabalin, selected according to the patient’s medical background, potential adverse effects, and drug interactions.^48^,^52^,^53^ Topical lidocaine or capsaicin may be considered for localized post-herpetic neuropathic pain when anatomically appropriate and well tolerated.

In suspected painful post-traumatic trigeminal neuropathy, early recognition is particularly important. Further unnecessary or irreversible dental treatment should be avoided once the pain is considered unlikely to arise from ongoing dental pathology. Patients should be referred to an orofacial pain specialist, oral and maxillofacial surgeon with expertise in trigeminal nerve injury, neurologist, or multidisciplinary pain service, depending on the suspected lesion and clinical setting.

Central neuropathic pain secondary to multiple sclerosis, stroke, or another central nervous system lesion generally requires neurologist- or pain-specialist-directed treatment. Management is often multidisciplinary and may combine pharmacological therapy, psychological support, rehabilitation, and treatment of the underlying neurological disorder. 48,49

Urgent neurological assessment and neuroimaging are required when facial pain is accompanied by acute focal neurological deficits, altered consciousness, sudden severe headache, progressive cranial nerve dysfunction, or other features suggestive of stroke or another acute central neurological process.

## 7. IDIOPATHIC AND NOCIPLASTIC OROFACIAL PAIN

Nociplastic pain is defined as pain arising from altered nociception despite no clear evidence of tissue damage causing peripheral nociceptor activation, nor evidence of somatosensory system disease or lesion.^6^ This category includes conditions that are mechanistically nociplastic yet diagnostically idiopathic.

### Persistent Idiopathic Facial Pain

Persistent idiopathic facial pain (PIFP) is persistent or recurrent facial or oral pain for which no underlying dental, neurological, or structural cause can be identified. The pain occurs daily or nearly daily for more than three months, is typically poorly localized, and does not follow the distribution of a peripheral nerve. It is usually described as dull, aching, or nagging, although burning or other qualities may also be reported. Clinical neurological examination is normal, and appropriate investigation has excluded dental and other local pathology. Unlike painful post-traumatic trigeminal neuropathy, PIFP is not associated with a demonstrable trigeminal nerve injury or objective sensory dysfunction.^56^,^57^

### Persistent Idiopathic Dentoalveolar Pain

Persistent idiopathic dentoalveolar pain (PIDP), previously termed atypical odontalgia, is persistent or recurrent pain localized to a tooth, an edentulous alveolar site, or the surrounding dentoalveolar tissues, despite the absence of clinical or radiographic evidence of dental pathology. The pain typically occurs daily for more than three months and may be described as dull, aching, pressure-like, or burning. It is considered an idiopathic pain disorder when no preceding causative event or demonstrable nerve injury can be identified.^56^,^57^ Unlike painful posttraumatic trigeminal neuropathy, it does not follow a clearly injured trigeminal nerve distribution and is not ordinarily accompanied by objective signs of trigeminal nerve dysfunction. Because the pain may be mistakenly attributed to dental disease, affected patients are at risk of undergoing unnecessary and potentially pain-aggravating dental procedures.

### Fibromyalgia with Orofacial Predominance

Fibromyalgia with orofacial predominance is a chronic pain syndrome of nociplastic origin, characterized by widespread pain, fatigue, sleep disturbances, and general sensitivity to touch. It is more common among women and sometimes undiagnosed for years. In the facial and jaw area, the most common symptom is dull, bilateral muscle pain in masticatory muscles and sometimes jaw fatigue during speech or chewing. Patients report high sensitivity to palpation even at light intensity.^58^,^59^

### Burning Mouth Syndrome

Burning mouth syndrome is a persistent idiopathic oral pain disorder characterized by daily burning or dysesthetic sensations of the oral mucosa for more than three months, despite clinically normal-appearing mucosa and no identifiable local or systemic cause. Symptoms are usually bilateral and most commonly affect the anterior tongue, although the lips, palate, gingiva, or entire oral cavity may be involved. Subjective xerostomia and altered or metallic taste are common accompanying symptoms.^60^,^61^

Diagnosis is one of exclusion and requires careful oral examination and consideration of alternative causes such as candidiasis, mucosal disease, salivary hypofunction, nutritional deficiencies, diabetes, thyroid disease, Sjögren disease, medication effects, and local irritation. The pathophysiology is incompletely understood but likely involves neuropathic and nociplastic mechanisms. Management includes patient education, treatment of any contributing factors, avoidance of oral irritants, and specialist-directed therapy with topical or systemic agents used for neuropathic pain. Psychological or behavioral interventions may also be beneficial when symptoms substantially affect mood, sleep, or daily functioning.

### Initial Management

Management of PIFP and PIDP is often multidisciplinary. Pharmacological treatment may include tricyclic antidepressants, particularly amitriptyline or nortriptyline, with serotonin–norepinephrine reuptake inhibitors or gabapentinoids considered when first-line treatment is ineffective or poorly tolerated. Psychological interventions, behavioral therapy, pain education, and support in developing coping strategies may be beneficial, particularly when pain has a substantial effect on mood, sleep, or daily functioning. Treatment aims to reduce pain, restore function, and improve quality of life rather than necessarily achieve complete pain elimination. Further irreversible dental procedures should be avoided in the absence of identifiable dental pathology, as they are unlikely to relieve the pain and may aggravate or perpetuate it.

## 8. PRIMARY HEADACHE DISORDERS PRESENTING AS OROFACIAL PAIN

Certain primary headache disorders may present predominantly or exclusively in the orofacial region, without typical cranial headache localization. These presentations frequently lead patients to seek dental or temporomandibular evaluation and may result in misdiagnosis and unnecessary interventions. These conditions are characterized by episodic or chronic pain patterns, associated headache features, and absence of objective findings on dental or temporomandibular examination. The two most clinically relevant entities in this category are orofacial migraine and tension-type orofacial pain.^62^

### Orofacial Migraine

Orofacial migraine represents a variant of migraine in which pain is localized to the teeth, jaws, gingiva, or other facial regions rather than the cranial vault. Pain is typically unilateral, throbbing or pulsating in quality, and of moderate to severe intensity. Attacks may last from several hours to days (by definition 4 to 72 hours) and often recur episodically, although chronic forms may occur. Importantly, pain is not provoked by chewing, jaw movement, or palpation of the TMJ or masticatory muscles, despite patients frequently attributing symptoms to dental pathology.

Associated migrainous features are key diagnostic clues and may include nausea, photophobia, phonophobia, osmophobia, and worsening with physical activity. Some patients report autonomic symptoms such as facial flushing, nasal congestion, or tearing, which can further confuse the clinical picture. Dental examination and imaging are typically normal, and pain usually does not respond to local dental anesthesia.

When orofacial pain follows a migrainous temporal pattern, is associated with typical migraine features, and lacks a local structural explanation, the clinician should strongly consider orofacial migraine. Management follows standard migraine principles, including trigger identification, acute abortive therapy, pain diary, and preventive treatment when indicated. Referral to neurology or a headache specialist is appropriate, particularly in recurrent or treatment-resistant cases.^62^,^63^

### Tension-type Orofacial Pain

Tension-type orofacial pain corresponds to tension-type headache in which pain is perceived primarily in the facial, jaw, or perioral regions. Pain is usually bilateral, dull, pressing, or tightening in quality, and of mild to moderate intensity. Unlike migraine, it is not pulsatile and is not aggravated by routine physical activity. Patients often describe a constant “band-like” or diffuse pressure and tightness across facial or jaw regions.

This condition frequently overlaps clinically with masticatory muscle pain and TMD, but important distinctions exist. Palpation of masticatory muscles may reproduce discomfort but does not reveal focal trigger points or significant functional limitation. Jaw movements are preserved, and pain does not worsen specifically with chewing or jaw loading. Associated headache features such as photophobia or nausea are absent or minimal.

Tension-type orofacial pain is often associated with psychosocial stress, sleep disturbance, anxiety, or widespread pain syndromes. The diagnosis is clinical and based on exclusion of dental, articular, infectious, and neuropathic causes. Management focuses on patient education, stress reduction, sleep optimization, and conservative pharmacologic therapy. Recognition of this entity is critical to avoid overtreatment of presumed temporomandibular or dental pathology and to direct patients toward appropriate multidisciplinary care when symptoms persist. 62,64

## 9. SYSTEMIC DISEASES WITH OROFACIAL PAIN MANIFESTATIONS

Numerous systemic conditions may present with orofacial pain as an early or prominent manifestation. 5 Recognition is crucial as orofacial symptoms may represent the first opportunity for diagnosis and intervention.

### Giant Cell Arteritis

Giant cell arteritis (GCA) is an inflammatory vascular disease involving medium and large arteries, mainly in patients over 60 years. The typical manifestation includes new temporal pain, scalp tenderness, and sometimes visual disturbances. A particularly important manifestation is jaw claudication—pain and fatigue during chewing caused by ischemia of masticatory muscles. Jaw claudication in elderly patients is a powerful predictor for positive biopsy in GCA.^4^,^65^ Early diagnosis is vital for preventing vision-threatening complications.^65^

For suspected GCA, immediate action is required: if inflammatory markers (erythrocyte sedimentation rate, C-reactive protein) are elevated and clinical suspicion is high, high-dose corticosteroids should be initiated immediately, even before temporal artery biopsy.

### Rheumatoid Arthritis, Spondyloarthropathies, and Crystal Arthropathies

These inflammatory arthropathies may involve the TMJ, resulting in pain, stiffness, and progressive functional impairment.

Rheumatoid arthritis most commonly causes bilateral TMJ involvement, presenting with preauricular pain, morning stiffness, restricted mouth opening, joint tenderness, and crepitus. Advanced disease may lead to condylar erosion, loss of ramus height, anterior open bite, or mandibular retrusion.

Spondyloarthropathies, including ankylosing spondylitis and psoriatic arthritis, may also affect the TMJ, although less frequently, producing inflammatory pain and limitation of mandibular movement.

Crystal arthropathies, particularly calcium pyrophosphate deposition disease (CPPD) and, less commonly, gout, may rarely involve the TMJ, presenting as acute monoarthritis with severe pain, swelling, and restricted jaw function that can mimic septic arthritis or internal derangement.

Recognition of inflammatory TMJ disease is important, particularly in patients with bilateral symptoms, prolonged morning stiffness, involvement of other joints, or unexplained occlusal changes, as these findings should prompt rheumatologic evaluation and early initiation of disease-modifying therapy to prevent irreversible joint destruction.^66^,^67^ For suspected systemic inflammatory arthritides, management should focus on prompt recognition, symptomatic relief, and timely referral to a rheumatologist for definitive diagnosis and initiation of disease-modifying therapy.

Treatment of the underlying disease typically involves disease-modifying antirheumatic drugs (DMARDs), biologic agents, or targeted synthetic therapies, depending on the specific diagnosis and disease severity. While awaiting rheumatologic evaluation, patients may benefit from conservative management of TMJ symptoms—including a soft diet, limitation of excessive jaw function, NSAIDs, and other supportive measures—which may provide symptomatic relief.^68^–^70^

### Systemic Lupus Erythematosus

Lupus is a multi-system autoimmune disease, common mainly in young women. In the head–neck area, manifestations may include bilateral TMJ pain (usually without structural deformities), recurring oral ulcers, or dry sensation. The physician may notice articular pain unexplained by regular imaging, accompanied by mouth dryness, raising suspicion of systemic disease requiring rheumatological evaluation.^71^ Patients presenting these symptoms should be referred to a rheumatologist for confirmation of the diagnosis and initiation of systemic immunomodulatory therapy. In the interim, orofacial symptoms may be managed conservatively with analgesics or NSAIDs, appropriate treatment of oral ulcers, and supportive measures for xerostomia.

### Systemic Sclerosis

Systemic sclerosis is an autoimmune connective tissue disease with pathological collagen deposition in various tissues. Orofacial manifestations include facial skin stiffness, trismus due to masticatory muscle movement limitation, gradual mouth opening limitation, and occasionally articular TMJ pain and dry mucosa. The physician may be first to notice jaw movement limitation and mouth opening difficulty. 72 Patients presenting with these findings should be referred to a rheumatologist for diagnostic confirmation, assessment of systemic involvement, and initiation or adjustment of disease-modifying therapy. In the interim, conservative management may include analgesics or NSAIDs when appropriate, regular jaw-stretching and physiotherapy to preserve oral aperture, meticulous oral hygiene, and supportive treatment for xerostomia.

## 10. REFERRED OROFACIAL PAIN FROM DISTANT MEDICAL CONDITIONS

Several serious medical conditions may present with jaw pain as primary or prominent symptom, despite having no direct involvement of orofacial structures.^73^

### Cardiac Ischemia (Angina Pectoris)

During temporary heart muscle ischemia (angina pectoris), visceral pain fiber stimulation occurs, draining to somatic sensory pathways at upper spinal cord levels. This somatotopic reflection leads many patients to experience cardiac pain as originating in one or both jaws, teeth, neck, or ear. Research indicates that while chest pain is the hallmark of ischemia, craniofacial pain is the sole presenting symptom in approximately 6% of patients with acute coronary syndrome, and up to 30% report radiation to jaw or neck.^74^,^75^ Pain usually occurs during exertion or stress, passes with rest, and is not exacerbated by masticatory function or palpation.

When cardiac ischemia is suspected, immediate cardiovascular evaluation with electrocardiography, cardiac biomarkers (troponins), cardiovascular risk factor assessment, and cardiology consultation when indicated is essential. The urgency of recognizing cardiac causes of jaw pain cannot be overstated, as delayed diagnosis may result in serious morbidity or mortality.

### Carotid and Vertebral Artery Dissection

Dissection of the internal carotid or vertebral artery is an important and frequently missed cause of acute head, facial, and neck pain, particularly in younger patients, and may precede transient ischemic attack or ischemic stroke by hours to several days. Ipsilateral facial, orbital, jaw, or tooth pain accompanied by neck pain is a common early presentation, and pain is often the first symptom, preceding any ischemic event. Features that should raise suspicion include a partial Horner syndrome (ptosis and miosis), pulsatile tinnitus, and a history of recent neck trauma, manipulation, or sudden neck movement. Because the pain may mimic trigeminal neuralgia, TMD, or dental pathology, a high index of suspicion is essential; suspected dissection warrants urgent vascular imaging (CT or MR angiography) and immediate neurological referral, as timely antithrombotic treatment reduces the risk of stroke.^76^

### Cervical Spine (Cervicogenic) Referred Pain

Disorders of the upper cervical spine may refer pain to the face and jaw through the trigeminocervical complex, where afferents from the upper three cervical nerve roots (C1–C3) converge with trigeminal afferents on shared second-order neurons. This convergence allows nociception arising from the upper cervical joints, discs, or muscles to be perceived in the orbital, frontal, temporal, auricular, or temporomandibular regions, and the referral is bidirectional. Cervicogenic pain is typically side-locked, provoked or worsened by neck movement or sustained posture, and associated with reduced cervical range of motion; it may closely mimic temporomandibular or primary headache disorders. Recognition rests on eliciting a temporal and mechanical relationship to the cervical spine, and management is directed at the underlying cervical source.^77^

### Paraneoplastic and Distant Tumor Orofacial Pain

Orofacial pain may rarely arise from distant malignancies without direct metastasis to the head and neck. Lung and other thoracic tumors have been reported to present with persistent unilateral pain involving the jaw, ear, temporal region, or other parts of the face, despite an unremarkable local examination. The mechanism is incompletely understood but may involve referred pain through vagal afferent pathways or, less commonly, a paraneoplastic neuropathy affecting trigeminal sensory pathways.

Apical lung tumors, including Pancoast tumors, should be considered particularly when unexplained orofacial pain is accompanied by shoulder or scapular pain radiating along the medial arm, hand weakness, or ipsilateral Horner syndrome caused by involvement of the lower brachial plexus and sympathetic chain. Respiratory symptoms may be absent early, contributing to delayed diagnosis. Persistent, progressive, or treatment-resistant unilateral facial or jaw pain—especially in a patient with smoking history, weight loss, cough, shoulder or arm symptoms, or Horner syndrome—should therefore prompt consideration of a thoracic source and appropriate chest imaging. These presentations are uncommon and usually represent a diagnosis reached after more frequent local, neurological, and musculoskeletal causes have been excluded.^33^,^78^

### Understanding Pain Chronification: From Acute to Chronic Orofacial Pain

Chronic pain is defined as pain persisting beyond three months, not resolving despite time elapsed since initial injury or treatment. This represents a condition where pain has lost its protective biological function and no longer reflects active or ongoing damage, but rather results from changes in nervous system function. Chronic orofacial pain may be continuation of acute pain (e.g. following trauma or treatment) or may appear independently without a clearly identifiable trigger.^56^,^57^

The transition from acute to chronic pain involves fundamental neuroplastic changes that alter pain processing at both peripheral and central nervous system levels. These mechanisms can affect any type of orofacial pain—whether originating from TMJ, masticatory muscles, nerves, or other structures—transforming initially localized, tissue-based pain into persistent, treatment-resistant conditions.

### Mechanisms of Chronification

#### Peripheral Sensitization

When tissue damage persists over time (e.g. chronic TMJ inflammation or recurring muscular pain), pain receptors (nociceptors) may develop low stimulus thresholds and increased response to normal stimuli. This peripheral sensitization leads to hypersensitivity and increased pain even without significant harmful stimulation. This pain remains of nociceptive origin but is accompanied by neurological hyperreaction making diagnosis and treatment difficult.^79^

#### Central Sensitization

Chronic pain conduction leads to central nervous system changes that amplify pain signals and create persistent pain states even after initial injury resolution. Key features include enhanced excitability of central pain pathways, reduced descending pain inhibition, altered neurotransmitter function, and structural changes in pain processing centers.^80^,^81^

### Nociplastic Pain Mechanisms

This represents chronic pain developing even in the absence of clear tissue or nerve damage, considered a result of central dysregulation in pain processing mechanisms. Pain persists, worsens, and may spread despite no pathological cause identifiable in examination or imaging. In these cases, neurological, emotional, and behavioral factors are involved.^82^

Examples of conditions with overlapping neuropathic and nociplastic mechanisms include burning mouth syndrome, atypical odontalgia, persistent idiopathic facial pain (PIFP), and chronic tension-type headache. Their clinical presentation suggests neuropathic mechanisms, yet in many cases no demonstrable nerve damage is found, pointing instead toward central sensitization and nociplastic pain processes. Comparable systemic conditions reflecting the same underlying mechanisms include fibromyalgia. 58,59

### Clinical Implications

Understanding chronification mechanisms has important clinical implications. Any acute orofacial pain condition—whether TMJ disorder, muscle dysfunction, or neuropathic pain—carries a risk of becoming chronic if not appropriately managed. Early intervention, addressing contributing factors (sleep disorders, psychological stress, parafunctional habits), and timely specialist referral when initial treatments fail are essential to prevent transition to chronic pain states.

Chronic orofacial pain typically responds less to conventional treatments and requires multidisciplinary approach combining pharmacological therapy with psychological-behavioral interventions to help patients cope with pain and modulate their response to it. The emphasis shifts from cure to functional restoration and improved quality of life.

### Sleep Disorders and Orofacial Pain

Sleep disturbance and chronic orofacial pain share a well-documented bidirectional relationship: poor or fragmented sleep lowers pain thresholds and impairs descending pain modulation, while persistent pain disrupts sleep, so that each perpetuates the other and contributes to chronification.^83^,^84^ Beyond sleep bruxism and sleep-disordered breathing, which act largely through the masticatory muscles (discussed above), insomnia and poor sleep quality are common in chronic orofacial pain and are associated with greater pain intensity and poorer treatment response; deterioration of sleep quality may even precede the onset of painful TMD.^85^ Obstructive sleep apnea deserves specific attention, as it is associated with increased nocturnal masticatory activity and morning craniofacial pain, and features such as snoring, witnessed apneas, daytime sleepiness, or obesity should prompt screening and sleep referral. 83,85 For these reasons, a brief sleep history—covering sleep quality, snoring and witnessed apneas, daytime sleepiness, and morning jaw symptoms—should form part of the assessment of any patient with chronic or recurrent orofacial pain, and treatment of coexisting sleep disorders should be integrated into the overall management plan.

## CONCLUSIONS

The differential diagnosis of orofacial and jaw pain represents a complex clinical challenge requiring systematic evaluation, comprehensive knowledge of diverse pathological processes, and appreciation for intricate anatomical relationships within the head and neck region. This review has presented a structured framework organizing potential non-odontogenic etiologies into ten clinically relevant categories, each with distinctive characteristics guiding diagnostic and therapeutic decision-making.

The systematic approach outlined emphasizes several crucial principles. The primacy of detailed history-taking and thorough physical examination cannot be overstated. While advanced imaging and laboratory studies play important roles in confirming diagnoses, the foundation of accurate assessment lies in careful clinical evaluation. The patient’s description of pain characteristics, temporal patterns, functional relationships, and associated symptoms provides the most valuable diagnostic information.

Recognition of red flag symptoms requiring urgent evaluation or immediate specialist referral is essential for optimal patient care. Conditions such as temporal arteritis, space-occupying lesions, and cardiac ischemia presenting with jaw pain may be life-threatening if diagnosis is delayed. Healthcare providers must maintain high clinical suspicion for these conditions and have a low threshold for urgent evaluation when clinical presentations suggest serious underlying pathology.

The multidisciplinary nature of orofacial pain management necessitates effective communication and collaboration between healthcare providers across multiple specialties. Primary care physicians, emergency medicine specialists, neurologists, rheumatologists, oral and maxillofacial surgeons, and other specialists must work together to provide comprehensive patient care. Clear understanding of appropriate referral patterns and specialist expertise enhances patient outcomes while ensuring efficient healthcare delivery.

The classification system presented provides a practical framework applicable across diverse clinical settings. Whether in primary care offices, emergency departments, or specialty clinics, this systematic approach enables healthcare providers to organize clinical thinking, guide appropriate investigations, and make informed management decisions. The emphasis on clinical assessment techniques accessible to physicians across various healthcare settings makes this framework broadly applicable.

## Abbreviations

BMS: burning mouth syndrome
CT: computed tomography
ENT: ear, nose, and throat
MRI: magnetic resonance imaging
NSAIDs: non-steroidal anti-inflammatory drugs
PET-CT: positron emission tomography-computed tomography
TMD: temporomandibular disorders
TMJ: temporomandibular joint
US: ultrasonography
